# Calcareous Degeneration of the Pericardium

**Published:** 1868-05-15

**Authors:** Curtis T. Fenn

**Affiliations:** Chicago


### CALCAREOUS DEGENERATION OF THE PERICARDIUM.

BY CURTIS T. FENN, M.D., CHICAGO.

THE following history was read in the Chicago Medical Society, and by request of the Society is sent to the JOURNAL:

James Owens, a laborer, sixty-five years old, born in Ireland, was admitted to the County Hospital Dec. 19, 1867. His complexion was sallow, eyes dark, with well marked arcus senilis. His feet and legs were œdematous and cyanotic, body emaciated, and remaining teeth worn, and free from decay.

After being carried to his bed, he assumed a semi-recumbent posture, keeping his left side a little raised. His circulation was always excitable, irregular, intermittent, and feeble. The radial pulse was felt with difficulty, owing to what seemed to be an obstruction in the walls of the artery. The apex beat of the heart was perceptible an inch to the left of its normal place; but there was a total want of rhythm, and a sense of distance in the sound. No friction sounds or murmurs were detected. Respiration was irregular, sighing, and imperfect, especially at night. His mental faculties were disturbed, passive delirium being generally manifest. His words were always incoherent, articulated in a mumbling way, so that rarely a word was intelligible. No paralysis was detected. He seemed often to have pain in his head. The pupil was contracted. The tongue was usually dry, brown, trembling, and pointed; appetite feeble; bowels constipated; urine normal in quantity, transparent, specific gravity 1012, reaction slightly acid, containing albumen which when coagulated formed a deposit equal to one fourth the whole volume. Nothing reliable was learned of his antecedent history, except the statement of his daughter, that he had once had rheumatism.

He was put upon a half ounce of gin, fifteen drops of *Tincture of chloride of iron*, and five grains of *Iodide of potassium*, three times daily. No amelioration of any of the symptoms followed. The patient died Feb. 2, 1868.

Autopsy forty-eight hours after death:

Emaciation was marked. The lungs were full of dark blood. Some old adhesions of the right pleura existed, and the middle lobe of the right lung was hepatized. The coverings of the heart in situ appeared normal, until an attempt was made to cut into the pericardium. Within its tissue was found an extensive deposit of calcareous matter, which covered the whole anterior face of the heart, and about half of the posterior. It spread out like a shell, being thickest where the pericardium unites with the central tendon of the diaphragm and gradually becoming thinner as it extended upward from the base in two parts, resembling the bivalve of an oyster, the distance of four and a half inches anteriorly, and three and a half posteriorly; the deposit was deficient over the upper part of the left margin of the heart, and extended to the right a little beyond the ventricular septum. A firm adhesion existed between the apex of the heart and base of the sack. This was severed, and the heart removed without injury to the shell. It maintained then the shape of a cup, somewhat irregular in outline, but capable of holding half a pint. The inner surface of the pericardium was roughened in spots by the concretion; otherwise both surfaces were smooth. The heart was enlarged and softened, and covered with a pearly deposit of plastic lymph. The right auricle and ventricle were filled with coagulum, and dilated. The tricuspid and mitral valves were calcified along their free border, but not impaired as to their sufficiency. The arch of the aorta, about the opening of the left subclavian, presented a roughened and calcified surface a square inch in area. Plastic deposits appeared along the inner surface of the thoracic aorta. The liver was enlarged and indurated, as if from chronic inflammation of the capsule; the gall bladder distended with black fluid. The kidneys were both atrophied to about one third their normal volume; the pyramids and cortical substance appeared blended, and contained cysts varying in size from that of a hazel nut to that of a pin head. One bore a cicatrix, as though a cyst had ruptured on the surface.

An examination of the brain showed a deposit of serum within the subarchanoidian space, but no lymph, and no adhesions. There was calcareous degeneration of the internal carotid and vertebral arteries, with the branches forming the circle of Willis. The walls of the ophthalmic arteries also were hardened, and their calibre diminished by the same foreign deposits. These degenerate arteries were not saved. The heart, pericardium, and arch of the aorta, are preserved in the County Hospital Museum.

*February* 14, 1868.

